# Supplementary material for: Proteomic aging signatures across mouse organs and life stages
Source: EMBO J. 2025 Jul 15;44(16):4631–60. doi: 10.1038/s44318-025-00509-x (PMC12361549; doi:10.1038/s44318-025-00509-x)
Supplement: Supplementary file 2 — Appendix [file 44318_2025_509_MOESM2_ESM.pdf]

Appendix for

Proteomic aging signatures across mouse organs and life stages

Table of contents

- Appendix Figure S1... 2
- Appendix Figure S2... 4
- Appendix Figure S3... 6
- Appendix Figure S4... 8

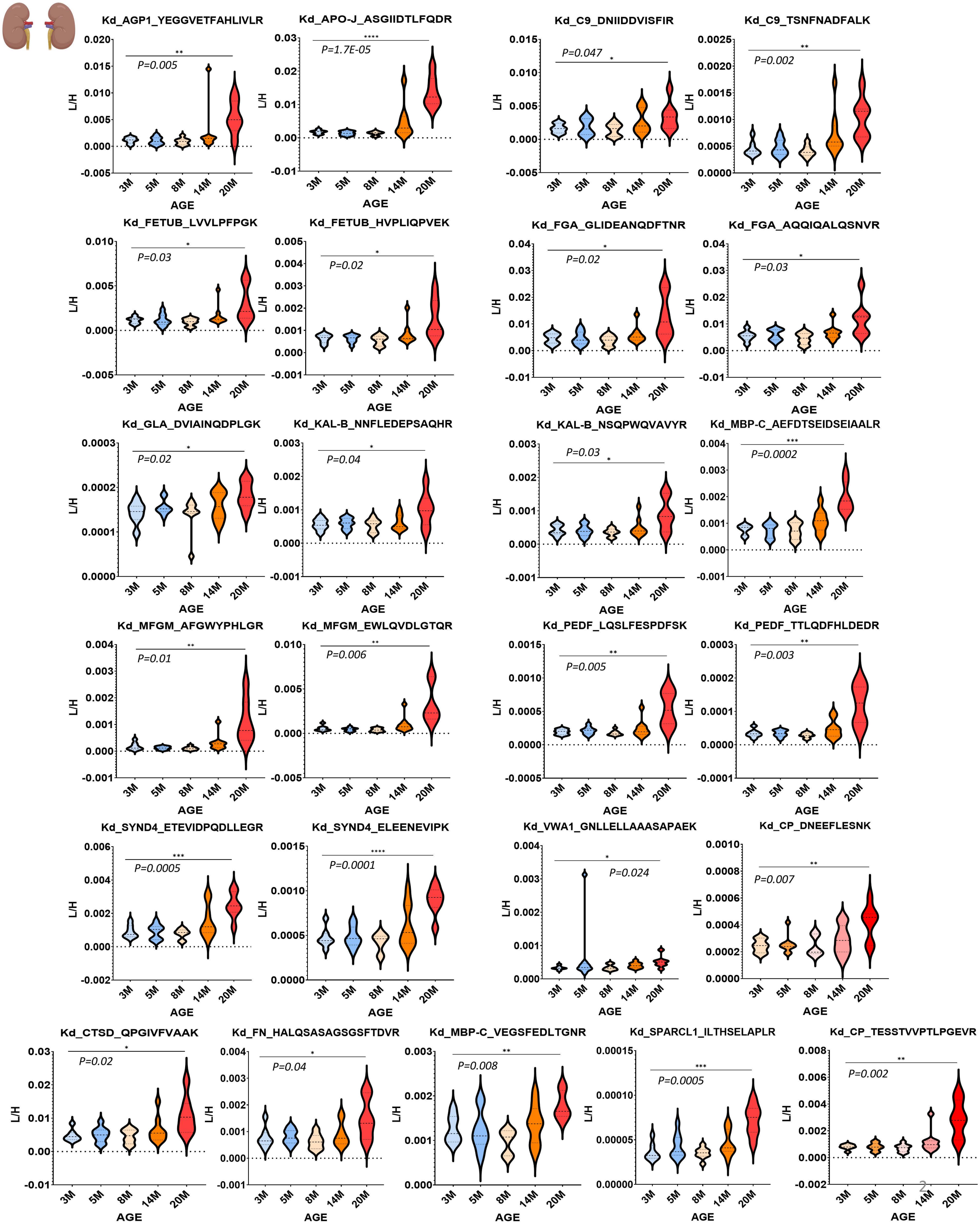
Kd_AGP1_YEGGVETFAHLIVLR
P=0.005
Kd_APO-J_ASGIIDTLFQDR
P=1.7E-05
Kd_C9_DNIIDDVISFIR
P=0.047
Kd_C9_TSNFNADFALK
P=0.002
Kd_FETUB_LVVLPFPGK
P=0.03
Kd_FETUB_HVPLIQPVEK
P=0.02
Kd_FGA_GLIDEANQDFTNR
P=0.02
Kd_FGA_AQQIQALQSNVR
P=0.03
Kd_GLA_DVIAINQDPLGK
P=0.02
Kd_KAL-B_NNFLEDEPSAQHR
P=0.04
Kd_KAL-B_NSQPWQVAVYR
P=0.03
Kd_MBP-C_AEFDTSEIDSEIAALR
P=0.0002
Kd_MFGM_AFGWYPHLGR
P=0.01
Kd_MFGM_EWLQVDLGTQR
P=0.006
Kd_PEDF_LQSLFESPDFSK
P=0.005
Kd_PEDF_TTLQDFHLDEDR
P=0.003
Kd_SYND4_ETEVIDPQDLLEGR
P=0.0005
Kd_SYND4_ELEENEVIPK
P=0.0001
Kd_VWA1_GNLLELLAAASAPAEK
P=0.024
Kd_CP_DNEEFLESNK
P=0.007
Kd_CTSD_QPGIVFVAAK
P=0.02
Kd_FN_HALQSASAGSGSFTDVR
P=0.04
Kd_MBP-C_VEGSFEDLTGNR
P=0.008
Kd_SPARCL1_ILTHSELAPLR
P=0.0005
Kd_CP_TESSTVVPTLPGEVR
P=0.002
L/H
AGE
3M
5M
8M
14M
20M

Appendix Figure S1. SureQuant-based quantitative mass spectrometry analysis of selected age-DEPs in aging mouse kidney. Comparison was performed using the 3 months old group as a reference and 7-9 biological replicates for each group. Mean light/heavy ratios (L/H) of unique peptides used for quantitation are depicted as violin plots. Bold dashed horizontal lines indicate medians, and lightly dashed lines indicate quartiles (*n* = 3). Statistical analysis was performed using the parametric unpaired one-sided t-test. The exact p values are shown in each figure. $P < 0.05$ is statistically significant. Abbreviations: Kd- kidney; 3, 5, 8, 14 & 20 M- denotes age in months. Source data are available online for this figure.

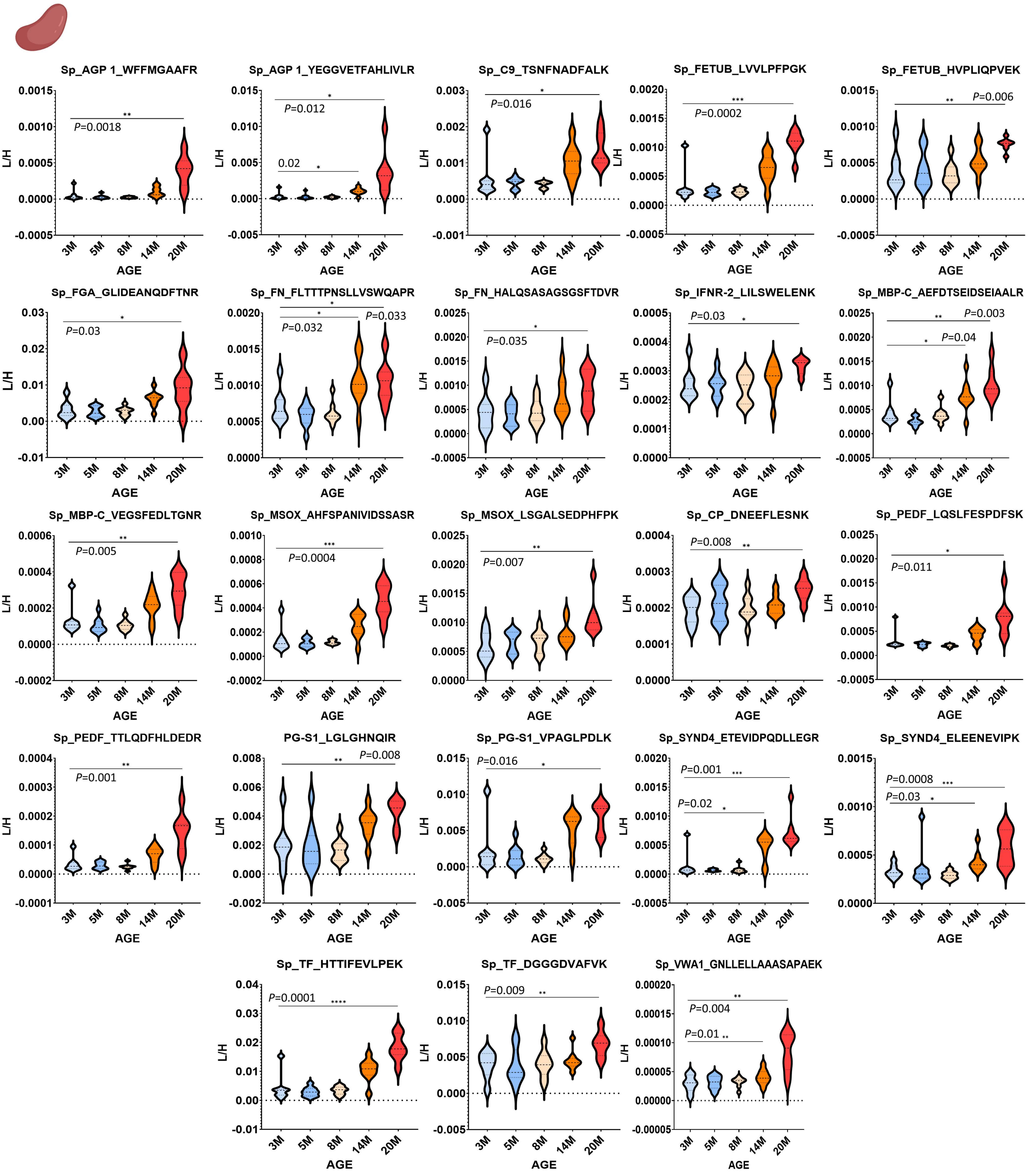
Sp_AGP 1_WFFMGAAFR
P=0.0018
Sp_AGP 1_YEGGVETFAHLIVLR
P=0.012
0.02
Sp_C9_TSNFNADFALK
P=0.016
Sp_FETUB_LVVLPFPGK
P=0.0002
Sp_FETUB_HVPLIQPVEK
P=0.006
Sp_FGA_GLIDEANQDFTNR
P=0.03
Sp_FN_FLTTTPNSLLVSWQAPR
P=0.032
P=0.033
Sp_FN_HALQSASAGSGSFTDVR
P=0.035
Sp_IFNR-2_LILSWELENK
P=0.03
Sp_MBP-C_AEFDTSEIDSEIAALR
P=0.003
P=0.04
Sp_MBP-C_VEGSFEDLTGNR
P=0.005
Sp_MSOX_AHFSPANIVIDSSASR
P=0.0004
Sp_MSOX_LSGALSEDPHFPK
P=0.007
Sp_CP_DNEEFLESNK
P=0.008
Sp_PEDF_LQSLFESPDFSK
P=0.011
Sp_PEDF_TTLQDFHLDEDR
P=0.001
PG-S1_LGLGHNQIR
P=0.008
Sp_PG-S1_VPAGLPDLK
P=0.016
Sp_SYND4_ETEVIDPQDLLEGR
P=0.001
P=0.02
Sp_SYND4_ELEENEVIPK
P=0.0008
P=0.03
Sp_TF_HTTIFEVLPEK
P=0.0001
Sp_TF_DGGGDVAFVK
P=0.009
Sp_VWA1_GNLLELLAAASAPAEK
P=0.004
P=0.01
L/H
AGE
3M
5M
8M
14M
20M

Appendix Figure S2. SureQuant-based quantitative mass spectrometry analysis of selected age-DEPs in aging mouse spleen. Comparison was performed using the 3 months old group as a reference and 7-9 biological replicates for each group. Mean light/heavy ratios (L/H) of unique peptides used for quantitation are depicted as violin plots. Bold dashed horizontal lines indicate medians, and lightly dashed lines indicate quartiles (n = 3). Statistical analysis was performed using the parametric unpaired one-sided t-test. The exact p values are shown in each figure. $P < 0.05$ is statistically significant. Abbreviations: Sp- spleen; 3, 5, 8, 14 & 20 M- denotes age in months. Source data are available online for this figure.

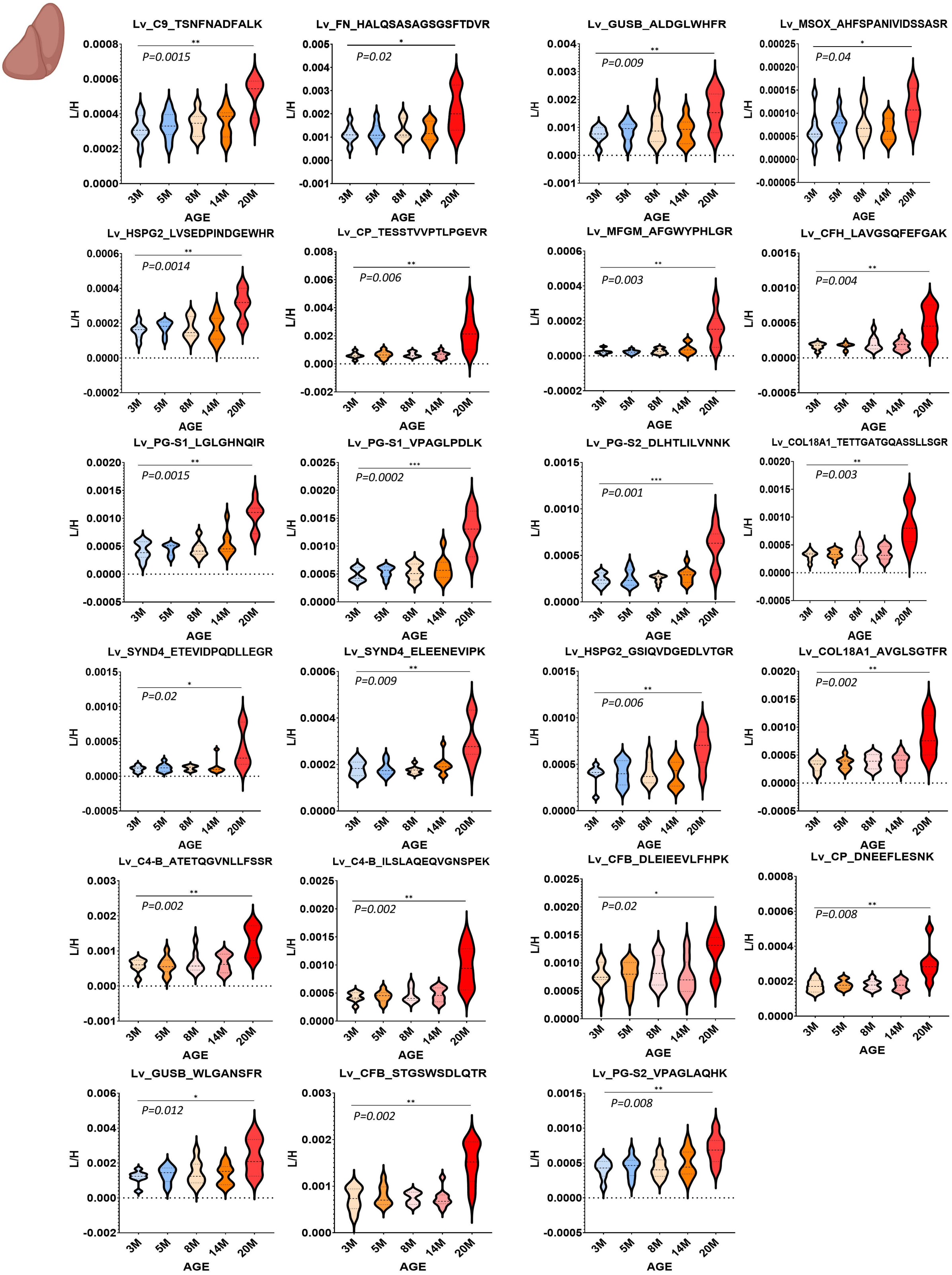
Lv_C9_TSNFNADFALK
P=0.0015
Lv_FN_HALQSASAGSGSFTDVR
P=0.02
Lv_GUSB_ALDGLWHFR
P=0.009
Lv_MSOX_AHFSPANIVIDSSASR
P=0.04
Lv_HSPG2_LVSEDPINDGEWHR
P=0.0014
Lv_CP_TESSTVVPTLPGEVR
P=0.006
Lv_MFGM_AFGWYPHLGR
P=0.003
Lv_CFH_LAVGSQFEFGAK
P=0.004
Lv_PG-S1_LGLGHNQIR
P=0.0015
Lv_PG-S1_VPAGLPDLK
P=0.0002
Lv_PG-S2_DLHTLILVNNK
P=0.001
Lv_COL18A1_TETTGATGQASSLLSGR
P=0.003
Lv_SYND4_ETEVIDPQDLLEGR
P=0.02
Lv_SYND4_ELEENEVIPK
P=0.009
Lv_HSPG2_GSIQVDGEDLVTGR
P=0.006
Lv_COL18A1_AVGLSGTFR
P=0.002
Lv_C4-B_ATETQGVNLLFSSR
P=0.002
Lv_C4-B_ILSLAQEQVGNSPEK
P=0.002
Lv_CFB_DLEIEEVLFHPK
P=0.02
Lv_CP_DNEEFLESNK
P=0.008
Lv_GUSB_WLGANSFR
P=0.012
Lv_CFB_STGSWSDLQTR
P=0.002
Lv_PG-S2_VPAGLAQHK
P=0.008
L/H
AGE
3M
5M
8M
14M
20M

Appendix Figure S3. SureQuant-based quantitative mass spectrometry analysis of selected age-DEPs in aging mouse liver. Comparison was performed using the 3 months old group as a reference and 7-9 biological replicates for each group. Mean light/heavy ratios (L/H) ratios of unique peptides used for quantitation are depicted as violin plots. Bold dashed horizontal lines indicate medians, and lightly dashed lines indicate quartiles (n = 3). Statistical analysis was performed using the parametric unpaired one-sided t-test. The exact p values are shown in each figure. $P < 0.05$ is statistically significant. Abbreviations: Lv- liver; 3, 5, 8, 14 & 20 M- denotes age in months. Source data are available online for this figure.

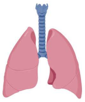

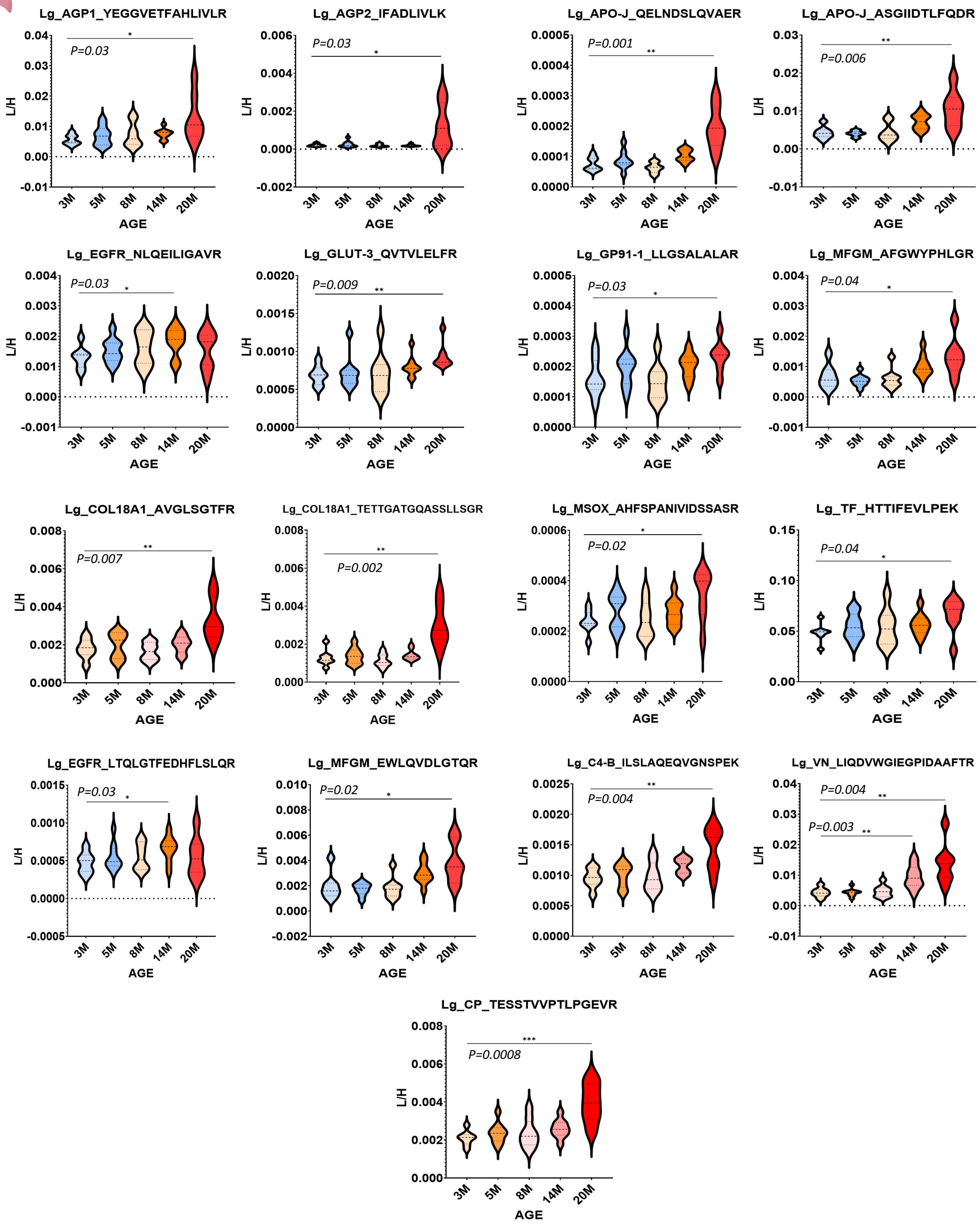

Lg_AGP1_YEGGVETFAHLIVLR
P=0.03
*
Lg_AGP2_IFADLIVLK
P=0.03
*
Lg_APO-J_QELNDSLQVAER
P=0.001
**
Lg_APO-J_ASGIIDTLFQDR
P=0.006
**
Lg_EGFR_NLQEILIGAVR
P=0.03
*
Lg_GLUT-3_QVTVLELFR
P=0.009
**
Lg_GP91-1_LLGSALALAR
P=0.03
*
Lg_MFGM_AFGWYPHLGR
P=0.04
*
Lg_COL18A1_AVGLSGTFR
P=0.007
**
Lg_COL18A1_TETTGATGQASSLLSGR
P=0.002
**
Lg_MSOX_AHFSPANIVIDSSASR
P=0.02
*
Lg_TF_HTTIFEVLPEK
P=0.04
*
Lg_EGFR_LTQLGTFEDHFLSLQR
P=0.03
*
Lg_MFGM_EWLQVDLGTQR
P=0.02
*
Lg_C4-B_ILSLAQEQVGNSPEK
P=0.004
**
Lg_VN_LIQDVWGIEGPIDAAFTR
P=0.004
**
P=0.003
**
Lg_CP_TESSTVVPTLPGEVR
P=0.0008
***
L/H
AGE
3M
5M
8M
14M
20M

Appendix Figure S4. SureQuant-based quantitative mass spectrometry analysis of selected age-DEPs in aging mouse lung. Comparison was performed using the 3 months old group as a reference and 7-9 biological replicates for each group. Mean light/heavy ratios (L/H) of unique peptides used for quantitation are depicted as violin plots. Bold dashed horizontal lines indicate medians, and lightly dashed lines indicate quartiles (*n* = 3). Statistical analysis was performed using the parametric unpaired one-sided t-test. The exact p values are shown in each figure. $P < 0.05$ is statistically significant. Abbreviations: Lg- lung; 3, 5, 8, 14 & 20 M- denotes age in months. Source data are available online for this figure.
