# Supplementary material for: Proteomic aging signatures across mouse organs and life stages
Source: EMBO J. 2025 Jul 15;44(16):4631–60. doi: 10.1038/s44318-025-00509-x (PMC12361549; doi:10.1038/s44318-025-00509-x)
Supplement: Supplementary file 22 — Expanded View Figures [file 44318_2025_509_MOESM22_ESM.pdf]

Expanded View Figures

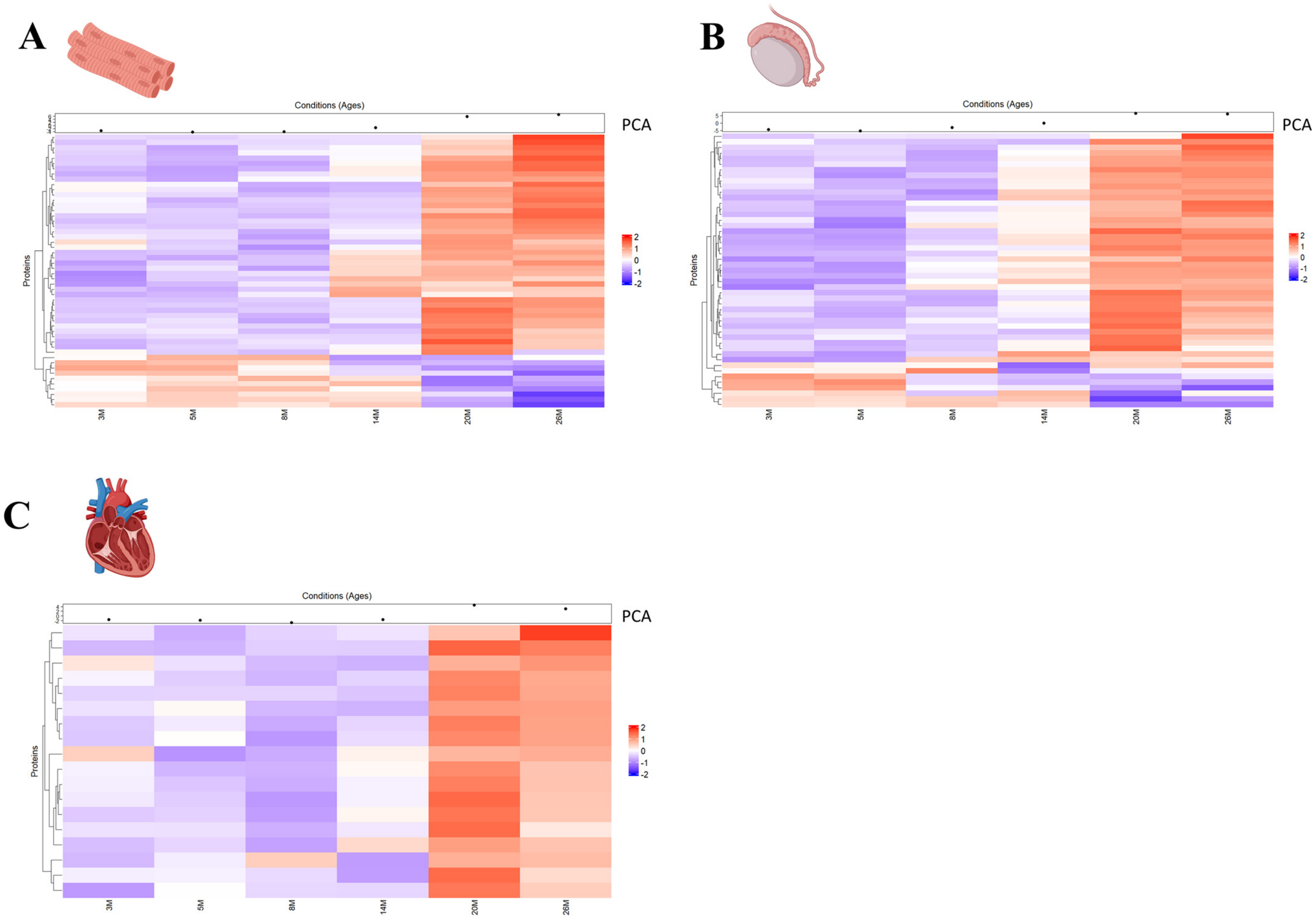

Figure EV1. Aging-associated protein expression changes in skeletal muscle, testis, and heart.

(**A–C**) Heatmaps illustrate differentially expressed proteins (DEPs) across six age groups (3, 5, 8, 14, 20, and 26 months) for skeletal muscle (**A**), testis (**B**), and heart (**C**). Proteins (rows) are clustered by expression trends, while columns represent age groups. The color gradient reflects log-transformed expression values, with red indicating higher expression (upregulation) and blue indicating lower expression (downregulation) relative to each protein's average level. Line graphs adjacent to the heatmaps depict the mean z-scored expression trajectories over time, with the *x* axis representing age and the *y* axis showing expression changes. Dot plots at the top highlight the principal component 1 (PC1) trajectories, capturing age-related shifts in protein expression. PC1 accounts for 80%, 79%, and 83% of the variance in skeletal muscle, testis, and heart, respectively. Source data are available on the Data Dryad public repository for this figure.

© The Author(s)

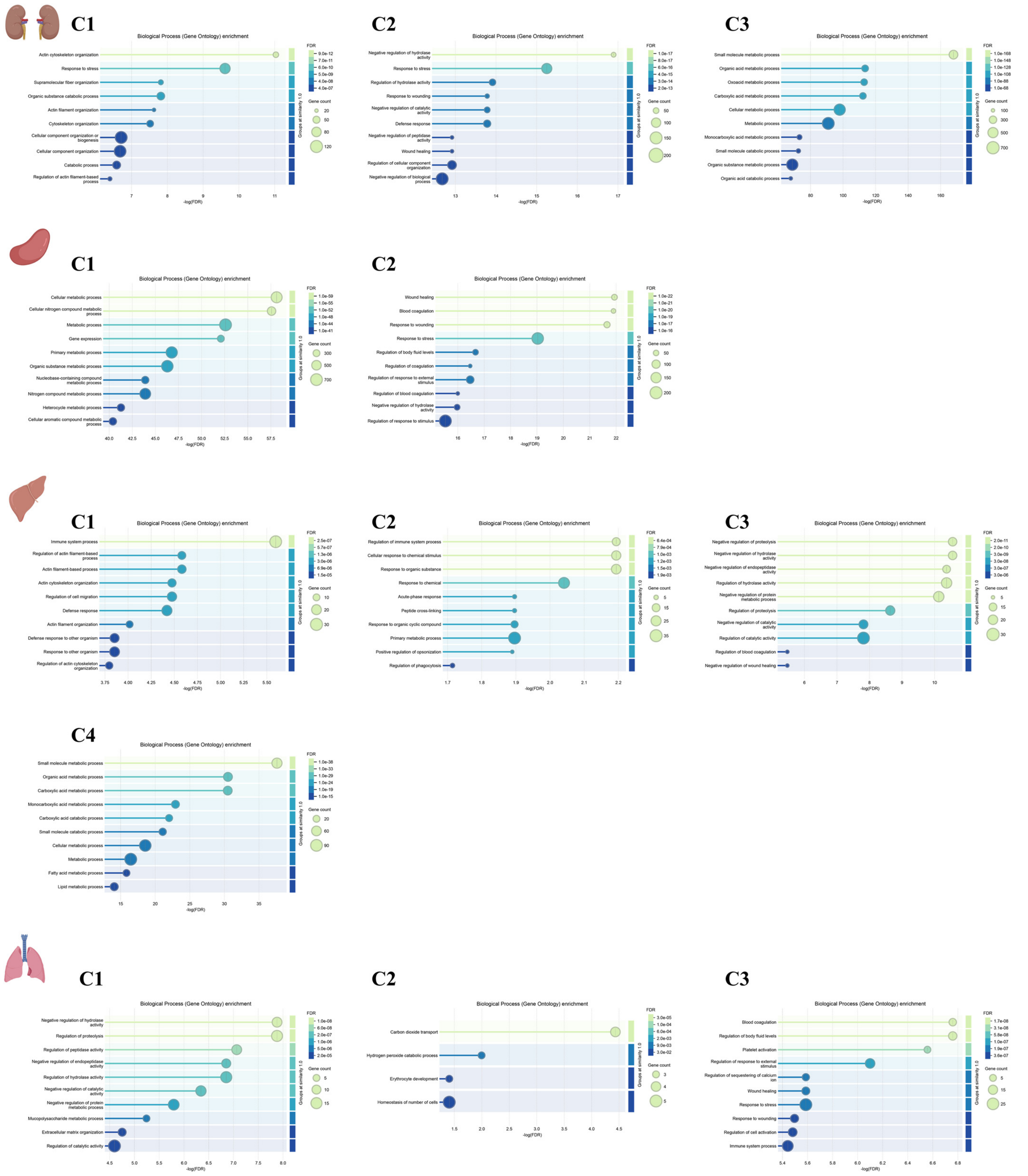

C1
Biological Process (Gene Ontology) enrichment
Actin cytoskeleton organization
Response to stress
Supramolecular fiber organization
Organic substance catabolic process
Actin filament organization
Cytoskeleton organization
Cellular component organization or biogenesis
Cellular component organization
Catabolic process
Regulation of actin filament-based process
-log(FDR)
C2
Biological Process (Gene Ontology) enrichment
Negative regulation of hydrolase activity
Response to stress
Regulation of hydrolase activity
Response to wounding
Negative regulation of catalytic activity
Defense response
Negative regulation of peptidase activity
Wound healing
Regulation of cellular component organization
Negative regulation of biological process
-log(FDR)
C3
Biological Process (Gene Ontology) enrichment
Small molecule metabolic process
Organic acid metabolic process
Oxoacid metabolic process
Carboxylic acid metabolic process
Cellular metabolic process
Metabolic process
Monocarboxylic acid metabolic process
Small molecule catabolic process
Organic substance metabolic process
Organic acid catabolic process
-log(FDR)
C1
Biological Process (Gene Ontology) enrichment
Cellular metabolic process
Cellular nitrogen compound metabolic process
Metabolic process
Gene expression
Primary metabolic process
Organic substance metabolic process
Nucleobase-containing compound metabolic process
Nitrogen compound metabolic process
Heterocycle metabolic process
Cellular aromatic compound metabolic process
-log(FDR)
C2
Biological Process (Gene Ontology) enrichment
Wound healing
Blood coagulation
Response to wounding
Response to stress
Regulation of body fluid levels
Regulation of coagulation
Regulation of response to external stimulus
Regulation of blood coagulation
Negative regulation of hydrolase activity
Regulation of response to stimulus
-log(FDR)
C1
Biological Process (Gene Ontology) enrichment
Immune system process
Regulation of actin filament-based process
Actin filament-based process
Actin cytoskeleton organization
Regulation of cell migration
Defense response
Actin filament organization
Defense response to other organism
Response to other organism
Regulation of actin cytoskeleton organization
-log(FDR)
C2
Biological Process (Gene Ontology) enrichment
Regulation of immune system process
Cellular response to chemical stimulus
Response to organic substance
Response to chemical
Acute-phase response
Peptide cross-linking
Response to organic cyclic compound
Primary metabolic process
Positive regulation of opsonization
Regulation of phagocytosis
-log(FDR)
C3
Biological Process (Gene Ontology) enrichment
Negative regulation of proteolysis
Negative regulation of hydrolase activity
Negative regulation of endopeptidase activity
Regulation of hydrolase activity
Negative regulation of protein metabolic process
Regulation of proteolysis
Negative regulation of catalytic activity
Regulation of catalytic activity
Regulation of blood coagulation
Negative regulation of wound healing
-log(FDR)
C4
Biological Process (Gene Ontology) enrichment
Small molecule metabolic process
Organic acid metabolic process
Carboxylic acid metabolic process
Monocarboxylic acid metabolic process
Carboxylic acid catabolic process
Small molecule catabolic process
Cellular metabolic process
Metabolic process
Fatty acid metabolic process
Lipid metabolic process
-log(FDR)
C1
Biological Process (Gene Ontology) enrichment
Negative regulation of hydrolase activity
Regulation of proteolysis
Regulation of peptidase activity
Negative regulation of endopeptidase activity
Regulation of hydrolase activity
Negative regulation of catalytic activity
Negative regulation of protein metabolic process
Mucopolysaccharide metabolic process
Extracellular matrix organization
Regulation of catalytic activity
-log(FDR)
C2
Biological Process (Gene Ontology) enrichment
Carbon dioxide transport
Hydrogen peroxide catabolic process
Erythrocyte development
Homeostasis of number of cells
-log(FDR)
C3
Biological Process (Gene Ontology) enrichment
Blood coagulation
Regulation of body fluid levels
Platelet activation
Regulation of response to external stimulus
Regulation of sequestering of calcium ion
Wound healing
Response to stress
Response to wounding
Regulation of cell activation
Immune system process
-log(FDR)
FDR
Gene count
Groups at similarity 1.0

 © The Author(s)

Figure EV2. Gene Ontology annotation of individual enriched MFuzz clusters for age-DEPs including the blood-derived proteome in the kidney, spleen, liver, and lung.

Enriched GO biological processes for clusters 1- 3 (C1-C3) for the kidney, clusters 1 and 2 (C1-C2) for the spleen, clusters 1–4 (C1-C4) for the liver and clusters 1–3 (C1-C3) for the lung are shown. Age-DEPs were clustered into temporal expression patterns using MFuzz clustering. Clusters represent groups of proteins with similar expression trajectories over six age groups (3, 5, 8, 14, 20, and 26 months). Gene Ontology (GO) enrichment analysis was performed for biological processes using STRING. The bar plots show enriched GO terms for each cluster, with the *x* axis representing -log10(*P* value) of enrichment and the y axis listing the GO terms. Bubble size indicates the number of proteins associated with each term, while the bubble color gradient corresponds to the false discovery rate (FDR), ranging from low (light green) to high (dark blue). Source data are available on the Data Dryad public repository for this figure.

© The Author(s)

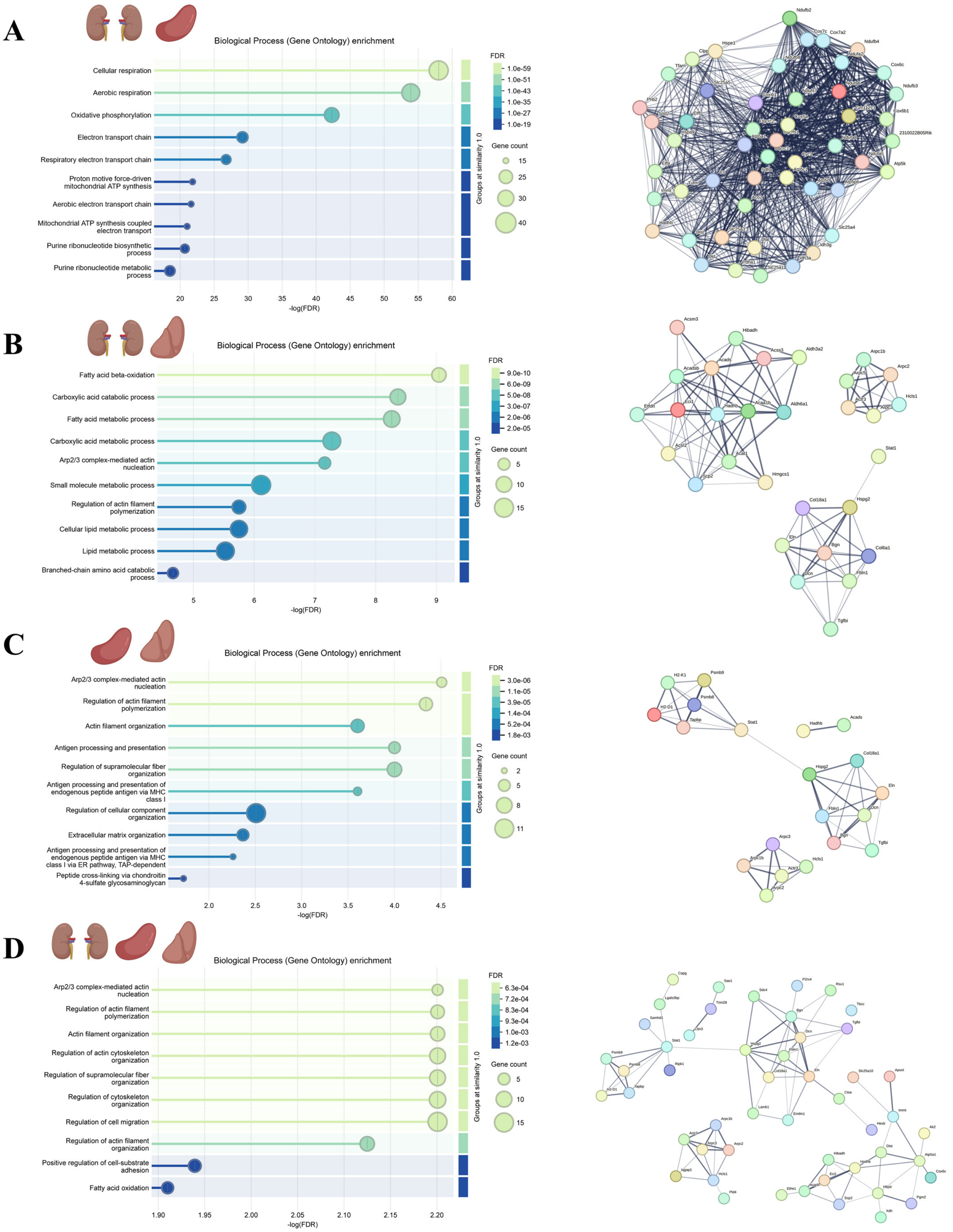
A
Biological Process (Gene Ontology) enrichment
Cellular respiration
Aerobic respiration
Oxidative phosphorylation
Electron transport chain
Respiratory electron transport chain
Proton motive force-driven mitochondrial ATP synthesis
Aerobic electron transport chain
Mitochondrial ATP synthesis coupled electron transport
Purine ribonucleotide biosynthetic process
Purine ribonucleotide metabolic process
20 25 30 35 40 45 50 55 60
-log(FDR)
Groups at similarity 1.0
FDR
1.0e-59
1.0e-51
1.0e-43
1.0e-35
1.0e-27
1.0e-19
Gene count
15
25
30
40
B
Biological Process (Gene Ontology) enrichment
Fatty acid beta-oxidation
Carboxylic acid catabolic process
Fatty acid metabolic process
Carboxylic acid metabolic process
Arp2/3 complex-mediated actin nucleation
Small molecule metabolic process
Regulation of actin filament polymerization
Cellular lipid metabolic process
Lipid metabolic process
Branched-chain amino acid catabolic process
5 6 7 8 9
-log(FDR)
Groups at similarity 1.0
FDR
9.0e-10
6.0e-09
5.0e-08
3.0e-07
2.0e-06
2.0e-05
Gene count
5
10
15
C
Biological Process (Gene Ontology) enrichment
Arp2/3 complex-mediated actin nucleation
Regulation of actin filament polymerization
Actin filament organization
Antigen processing and presentation
Regulation of supramolecular fiber organization
Antigen processing and presentation of endogenous peptide antigen via MHC class I
Regulation of cellular component organization
Extracellular matrix organization
Antigen processing and presentation of endogenous peptide antigen via MHC class I via ER pathway, TAP-dependent
Peptide cross-linking via chondroitin 4-sulfate glycosaminoglycan
2.0 2.5 3.0 3.5 4.0 4.5
-log(FDR)
Groups at similarity 1.0
FDR
3.0e-06
1.1e-05
3.9e-05
1.4e-04
5.2e-04
1.8e-03
Gene count
2
5
8
11
D
Biological Process (Gene Ontology) enrichment
Arp2/3 complex-mediated actin nucleation
Regulation of actin filament polymerization
Actin filament organization
Regulation of actin cytoskeleton organization
Regulation of supramolecular fiber organization
Regulation of cytoskeleton organization
Regulation of cell migration
Regulation of actin filament organization
Positive regulation of cell-substrate adhesion
Fatty acid oxidation
1.90 1.95 2.00 2.05 2.10 2.15 2.20
-log(FDR)
Groups at similarity 1.0
FDR
6.3e-04
7.2e-04
8.3e-04
9.3e-04
1.0e-03
1.2e-03
Gene count
5
10
15

 © The Author(s)

Figure EV3. Gene Ontology annotation and STRING network analysis of shared non-blood-derived age-DEPs in 2 or more organs.

Enriched GO biological processes and STRING interaction network clusters for the top hub proteins identified in the shared age-DEPs between the kidney and spleen (**A**), kidney and liver (**B**), spleen and liver (**C**) as well as kidney, spleen and liver (**D**) are shown. Left Panels: GO plots showing enriched biological processes for the shared age-DEPs. The significance of enrichment is indicated on the x axis with the -log10(FDR), while protein count and false discovery rate (FDR), are depicted by the size and color of circles, respectively. Right Panels: STRING interaction networks for the top hub proteins identified using Cytoscape's CytoHubba. Each node represents a protein, and edges indicate functional associations. Source data are available on the Data Dryad public repository for this figure.

© The Author(s)

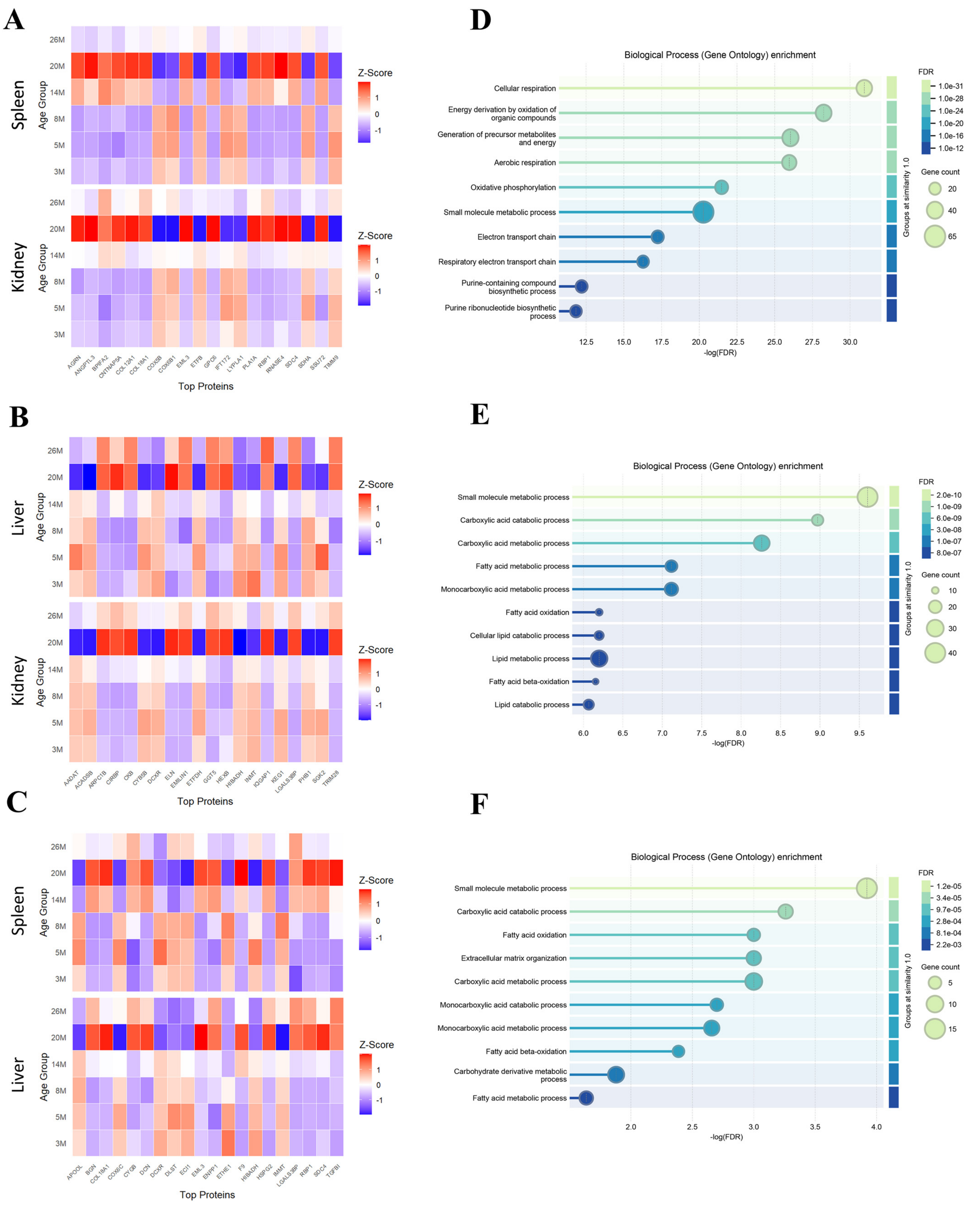
A
Spleen
Kidney
Age Group
26M
20M
14M
8M
5M
3M
Z-Score
1
0
-1
AGRN ANGPTL3 BPIFA2 CNTNAP5A COL12A1 COL18A1 COX5B COX6B1 EML3 ETFB GPC6 IFT172 LYPLA1 PLA1A RBP1 RNASE4 SDC4 SDHA SSU72 TIMM9
Top Proteins
B
Liver
Kidney
AADAT ACADSB ARPC1B CIRBP CKB CYB5B DCXR ELN EMILIN1 ETFDH GGT5 HEXB HIBADH INMT IQGAP1 KEG1 LGALS3BP PHB1 SGK2 TRIM28
Top Proteins
C
Spleen
Liver
APOOL BGN COL18A1 COX6C CYGB DCN DCXR DLST ECI1 EML3 ENPP1 ETHE1 F9 HIBADH HSPG2 IMMT LGALS3BP RBP1 SDC4 TGFBI
Top Proteins
D
Biological Process (Gene Ontology) enrichment
Cellular respiration
Energy derivation by oxidation of organic compounds
Generation of precursor metabolites and energy
Aerobic respiration
Oxidative phosphorylation
Small molecule metabolic process
Electron transport chain
Respiratory electron transport chain
Purine-containing compound biosynthetic process
Purine ribonucleotide biosynthetic process
12.5 15.0 17.5 20.0 22.5 25.0 27.5 30.0
-log(FDR)
Groups at similarity 1.0
FDR
1.0e-31
1.0e-28
1.0e-24
1.0e-20
1.0e-16
1.0e-12
Gene count
20
40
65
E
Biological Process (Gene Ontology) enrichment
Small molecule metabolic process
Carboxylic acid catabolic process
Carboxylic acid metabolic process
Fatty acid metabolic process
Monocarboxylic acid metabolic process
Fatty acid oxidation
Cellular lipid catabolic process
Lipid metabolic process
Fatty acid beta-oxidation
Lipid catabolic process
6.0 6.5 7.0 7.5 8.0 8.5 9.0 9.5
-log(FDR)
Groups at similarity 1.0
FDR
2.0e-10
1.0e-09
6.0e-09
3.0e-08
1.0e-07
8.0e-07
Gene count
10
20
30
40
F
Biological Process (Gene Ontology) enrichment
Small molecule metabolic process
Carboxylic acid catabolic process
Fatty acid oxidation
Extracellular matrix organization
Carboxylic acid metabolic process
Monocarboxylic acid catabolic process
Monocarboxylic acid metabolic process
Fatty acid beta-oxidation
Carbohydrate derivative metabolic process
Fatty acid metabolic process
2.0 2.5 3.0 3.5 4.0
-log(FDR)
Groups at similarity 1.0
FDR
1.2e-05
3.4e-05
9.7e-05
2.8e-04
8.1e-04
2.2e-03
Gene count
5
10
15

© The Author(s)

Figure EV4. Pairwise correlation analysis of shared age-DEPs in kidney and spleen, kidney and liver, liver and spleen.

Heatmaps displaying the expression trajectories of highly correlated proteins (Spearman's correlation coefficient >0.5) across paired organ comparisons: (**A**) spleen and kidney, (**B**) kidney and liver, and (**C**) spleen and liver. The color gradient indicates the normalized and log-transformed expression values, ranging from low (blue) to high (red) relative to the mean (standard error of the mean (SEM) is 0.1, 0.18, and 0.11 for the kidney, spleen, liver). Each heatmap represents the top 20 positively correlated proteins for the corresponding organ pair. Age groups (in months, e.g., 3 M, 5 M) are displayed on the y axis, and proteins are listed on the *x* axis. (**D–F**) Gene Ontology (GO) biological process enrichment analysis in STRING for proteins positively correlated (Spearman's correlation coefficient >0.5) in each organ pair: (**D**) spleen-kidney, (**E**) kidney–liver, and (**F**) spleen–liver. The *x* axis represents the -log10(FDR) of the enriched terms, while the bubble size corresponds to the number of genes associated with each term. The color gradient reflects the significance of enrichment, with lighter and darker shades indicating lower and higher FDR values, respectively. Source data are available on the Data Dryad public repository for this figure.

© The Author(s)

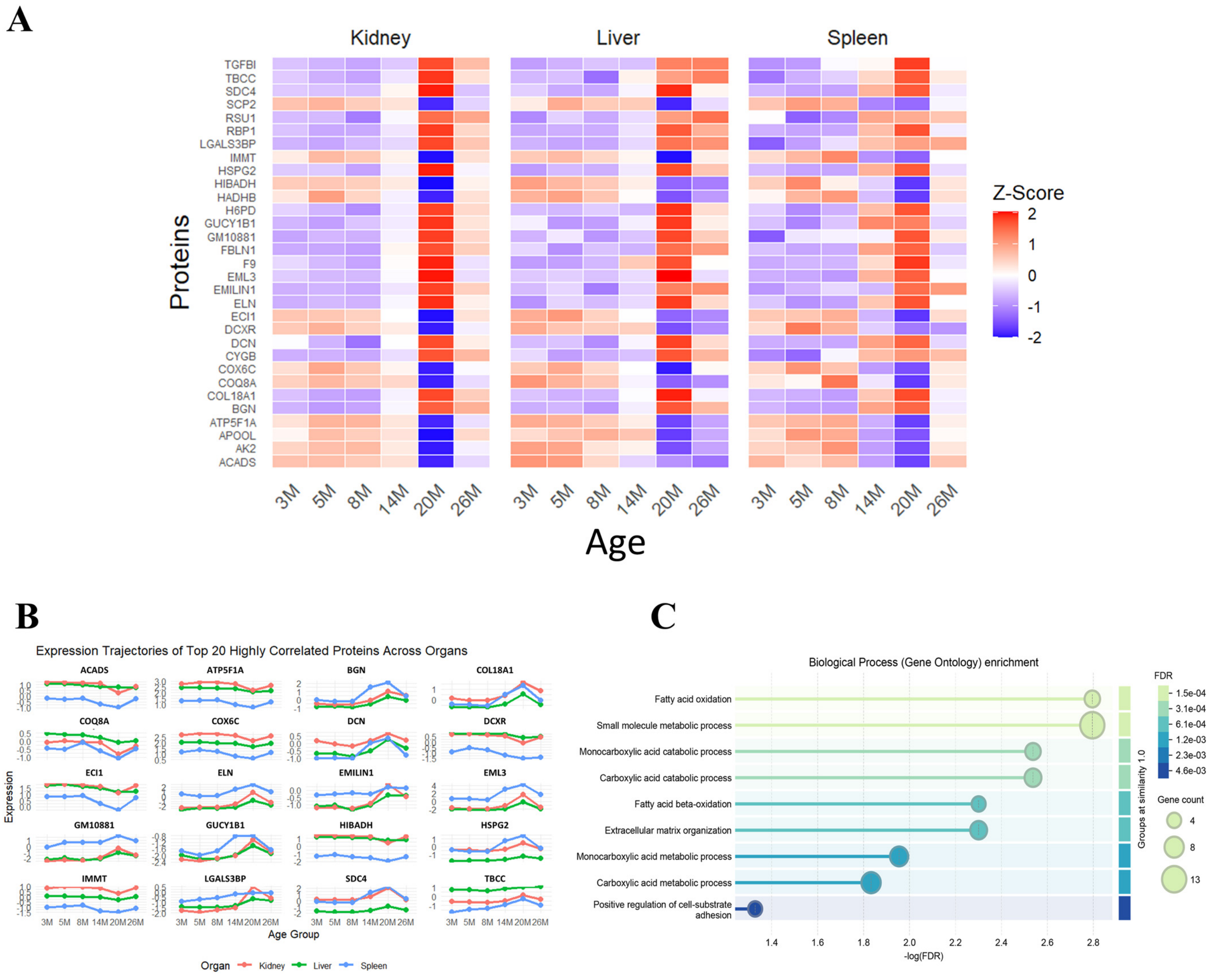

Figure EV5. Correlation analysis of shared age-DEPs in kidney, liver and spleen.

(**A**) Heatmaps showing the expression patterns of 31 highly correlated proteins (Spearman's correlation coefficient >0.5 across all pairwise organ comparisons) in kidney, liver, and spleen. The *x* axis represents the age groups (in months, e.g., 3 M, 5 M), while the y axis lists the proteins. The color gradient denotes normalized and log-transformed expression levels, with red indicating high expression and blue indicating low expression relative to the mean. (**B**) Line graphs depicting the expression trajectories of the top 20 highly correlated proteins across kidney, liver, and spleen. Each plot represents the protein expression trajectory across age groups for the three organs, providing a visual comparison of expression dynamics. The consistent trends across organs demonstrate the shared correlation of protein expression across the biological systems. (**C**) Gene Ontology (GO) enrichment analysis of the highly correlated proteins. The x axis represents the -log10(FDR) of enriched biological processes, while the bubble size reflects the number of proteins associated with each term. The color gradient indicates FDR significance, with lighter and darker shades denoting lower and higher significance, respectively. Source data are available on the Data Dryad public repository for this figure.

© The Author(s)
